# Disc degeneration is easily occurred at the same and adjacent cephalad level in cervical spine when Modic changes are present

**DOI:** 10.1186/s13018-023-04015-w

**Published:** 2023-07-31

**Authors:** Junhui Liu, Binhui Chen, Lu Hao, Zhi Shan, Yilei Chen, Fengdong Zhao

**Affiliations:** 1 Department of Orthopaedics, Sir Run Run Shaw Hospital, School of Medicine, Zhejiang University, No. 3, Qingchun Road East, Hangzhou, 310016 People’s Republic of China; 2 Key Laboratory of Musculoskeletal System Degeneration and Regeneration Translational Research of Zhejiang Province. No. 3, Qingchun Road East, Hangzhou, 310016 People’s Republic of China; 3 Department of Orthopaedic Surgery, Ningbo Medical Center Li Huili Hospital, Ningbo, Zhejiang People’s Republic of China

**Keywords:** Modic changes, Adjacent cephalad level, Disc degeneration, Cervical spine

## Abstract

**Objective:**

This research aimed to evaluate the influence of Modic changes (MCs) on disc degeneration at the same and adjacent cephalad levels in the cervical spine.

**Methods:**

This research retrospectively reviewed 1036 patients with neck pain, upper limb pain, or numbness who were treated at our out-patient clinic and underwent cervical MRI and cervical anteroposterior/lateral radiography from Jan, 2016 to Jan, 2021. MCs and disc degeneration parameters at same and nearby cephalad levels of MCs were evaluated. Discs were divided into the MCs, adjacent, and control groups, and the association between MCs and disc degeneration at the same and adjacent cephalad levels was investigated.

**Results:**

Of the 1036 patients whose MRI scans were reviewed, 986 met the inclusion criteria (503 women and 483 men; average age, 62.8 years; scope of 35–79 years). The prevalence of MCs in the cervical spine was 13.0% (128/986). Type I, II, III changes were observed in 38 (29.69%), 82 (64.06%), and 8 (6.25%) patients, respectively. MCs were most frequently identified at the C5–6 (59/986; 5.98%) and C6–7 (38/986; 3.85%) levels. Disc with MCs showed worse outcomes with regard to disc degeneration grade, anterior osteophyte formation than the adjacent and control groups (*p* < 0.05), whereas they were more severe in the adjacent group compared to normal group.

**Conclusion:**

Our findings indicate that MCs increased disc degeneration at the same and nearby cephalad levels in cervical spine, and the severity of degeneration at the same segment was more serious than that at the cephalad level.

## Introduction

Modic changes (MCs) mainly include the signal intensity changes at nearby vertebral bone marrow on MRI [1, 2]. Changes in these vertebral end-plate signals were first described by De Roos [3] in 1987, and Modic [2, 4] discussed the relevant change features. MCs can be classified into three types based on different characteristics: type I lesions involve low T1 and high T2 signals, which are associated with the continuous degeneration of vascularized fibrous tissue in bone marrow; Type II lesions involve high T1 and T2 signals, mainly reflecting the phenomenon of fat replacement in bone marrow; Type III lesions come last, involve low T1 and T2 signals, and their signal characteristics are correlated with endplate sclerosis [4].

Since the introduction of these changes by Modic in 1988 [2], many scholars researched the MCs of lumbar spine. But only few of them had evaluated MCs in cervical spine. Some studies have investigated the relationship between degenerative spine changes, such as disc displacement, Schmorl's nodes, and facet degeneration, and MCs and pain in the cervical spine [2, 5–7]. However, to the best of our knowledge, no study has investigated the relation between MCs and disc degeneration (DD) at the same and adjacent cephalad levels. Our preliminary experiment including 200 cases showed some correspondence between MCs and DD at the same and nearby cephalad levels. This paper aimed to confirm this conclusion in widely population. Therefore, in this study, the effect of MCs on disc degeneration and severity at the same and nearby cephalad levels in cervical spine were evaluated.

## Materials and methods

### Patients

We selected 1036 cases (with neck pain and discomfort or upper limb pain or numbness) who visited our out-patient clinic and underwent cervical MRI and cervical radiography from 2016.1 to 2021.1. The exclusion criteria are as follows: recent spinal surgery, spinal infection, inflammatory spondyloarthropathy, vertebral atresia, and chronic inflammation.

### Imaging evaluation

The presence, type, and location of MCs were assessed based on MRI scans, and DD parameters at the same and nearby cephalad levels of MCs were detected based on X-ray and MRI scans.

**Fig. 1:**
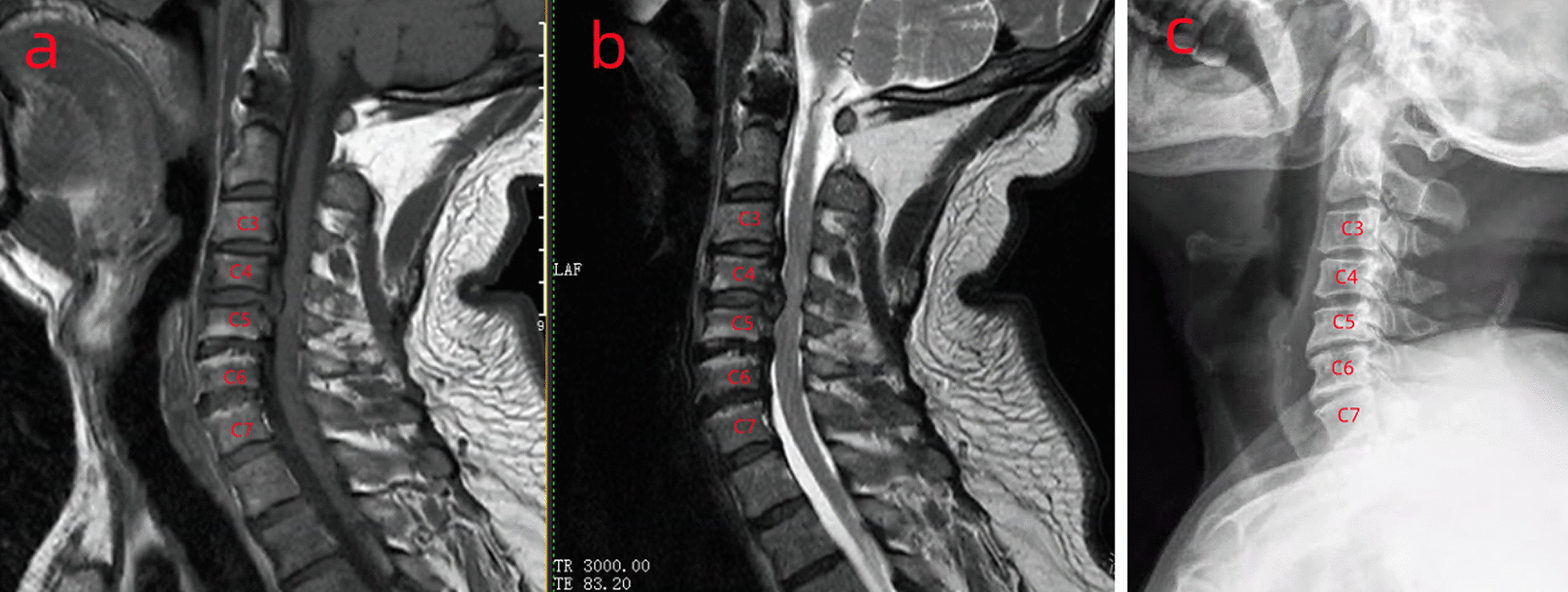
Patient; 60-year-old man, T1-weighted (**a**) and T2-weighted (**b**) magnetic resonance images of the cervical end-plates showing the presence of type II Modic changes (indicated by high signal on T1 and T2) on C5–6 level. C4–5 was defined as the adjacent cephalad level, and C3–4 with grade 2 was defined as the control. X-ray showed anterior osteophyte formation on C5–6 level (**c**)

Disc degeneration was evaluated according to the grading criteria for cervical DD (Table 1) [8, 9]; a disc with grade 1 or 2 in the same patient was defined as the control. The severity of disc degeneration at same and nearby cephalad levels was compared with that of the control. Discs were divided into the MCs, adjacent, and control groups, and the effect of MCs on disc degeneration at the same and adjacent cephalad levels was investigated (Fig. 1).

**Table 1:** Grading system for cervical intervertebral disc degeneration

Grade	Nucleus signal intensity	Nucleus structure	Distinction of nucleus and annulus	Disc height
1	Hyperintense	Homogeneous, white	Clear	Normal
2	Hyperintense	Inhomogeneous with horizontal band, white	Clear	Normal
3	Intermediate	Inhomogeneous, gray to black	Unclear	Normal to decreased
4	Hypointense	Inhomogeneous, gray to black	Lost	Normal to decreased
5	Hypointense	Inhomogeneous, gray to black	Lost	Collapsed

Imaging evaluation was performed by radiologist and spine surgeon of blinded condition. The consensus was reached under condition of divergence existed. The kappa index was 0.82.

### Disc degeneration parameters

Plain radiographic assessments included disc height and anterior osteophyte formation. The disc height was defined as the distance between the middle point of endplates. The disc height was measured using digital image processing software (Image J from National Institutes of Health, Bethesda, MD, USA). DD was also assessed based on the corresponding grading system [8, 9]. The reliability of this grading system has been previously reported [9] (Fig. 1).

The relevant research scheme was approved by the medical ethics committee of the hospital, and all the candidates were informed of the research situation and precautions, obtained their informed consent, entered the collected information, and kept in a unified and standardized manner to provide support for subsequent study.

### Data analysis

Data were analyzed using Excel (Microsoft, WA, USA) and SPSS20.0 software, and quantitative data were described as mean ± SD. Two-sided group t-tests were applied to determine the mean values difference of different groups. And *p* < 0.05 was regarded as the significant judgement standard.

## Results

### Prevalence of MCs at each vertebral level

Of the 1,036 patients whose MRI scans were reviewed, 986 met the inclusion criteria (503 women and 483 men; average age, 62.8 years; scope of 35–79 years). The prevalence of MCs in the cervical spine was 13.0% (128/986) in the 986 patients. Type II was the most commonly observed MCs, followed by type I, and type III was the least prevalent. Types I, II, and III changes were observed in 38 (29.69%), 82 (64.06%), and 8 (6.25%) patients, respectively. MCs were most frequently identified at the C5–6 (59/986; 5.98%) and C6–7 (38/986; 3.85%) levels; 53% of the MCs were observed in women, whereas 47% of the changes were evident in men, no obvious sex differences exist in different groups (*P* > 0.05).

### Effect of MCs on DD at the same and nearby cephalad levels

The effects of MCs on DD at the same and nearby cephalad levels were shown in Tables 2 and 3. Disc with MCs exhibit worse outcomes with regard to disc degeneration grade, anterior osteophyte formation than the adjacent and control groups (*p* < 0.05). Outcomes in the adjacent group were more severe compared to that of control group. MCs increased disc degeneration at same and nearby cephalad levels in the cervical spine, and degeneration was more severe at the same segment than that at the cephalad level.

**Table 2:** Comparison of disc height and anterior osteophyte formation between the modic changes (MCs), adjacent, and control groups

Group	Disc height (mm)	Anterior osteophyte (n = 128)
MCs group	4.23 ± 0.41	93/128
Adjacent group	5.67 ± 0.28	62/128
Control group	6.81 ± 0.19	12/128

**Table 3:** Comparison of disc degeneration grade between the Modic changes (MCs), adjacent, and control groups

Group	Grade 1/2	Grade 3	Grade 4	Grade 5
MCs group (n = 128)	0	18	66	44
Adjacent group (n = 128)	24	32	37	35
Control group (n = 128)	128	0	0	0

## Discussion

The present study investigated the effect of MCs on disc degeneration at same and nearby cephalad levels in cervical spine. Our study showed that the prevalence of MCs was 13.0%, with type II predominating. Consensus has been reached in this respect [10–13]. The result of this paper suggested that cervical segments with MCs were significantly more likely to have disc degeneration. In addition, MCs increased DD at nearby cephalad level in the cervical spine, and degeneration at the same segments was more severe than at the cephalad level, indicating that MCs may be an important sign of disc degeneration at the same and adjacent cephalad levels.

### Effect of MCs on disc degeneration at the same level

Hayashi found that patients with MCs had severe DD compared to those without, which may cause range of motion reduced [10]. Our study found that such kind of patients had worse outcomes in regard to disc degeneration grade, disc space height, and anterior osteophyte formation, which was also consistent with the study by Mann et al. who found a link between MCs and disc herniation [14]. In our study, we found that MCs possibly cause and promote disc degeneration, one explanation is because MCs can destroy the structure of intervertebral disc and endplate, and increase the abnormal loading on disc [15, 16]. Besides, the nutritional pathway associated with the disc will also be inhibited. All these confirmed a conclusion that MCs are possible causes of DD, and they may promote degeneration when the disc has slight aging degeneration.

### Effect of MCs on DD at the nearby cephalad level

According to relevant report, no study has assessed the relationship between DD at the nearby cephalad level and MCs in cervical spine. In our research, it can be inferred that discs with MCs were significantly more prone to have DD and a lower disc height, indicating that the disc maintains a relatively steady state, which may affect the loading conditions at the adjacent motor units. The mobility of the cervical spine is then transferred to nearby segment, accelerating the early development of degenerative changes in the disc above, which may explain our finding.

Our study had several limitations. First, it belongs to retrospective research. Second, it only included symptomatic cases, whereas healthy volunteers were not included, which may have limited the accuracy of the results. Moreover, small sample inhibits us from determining the effect of MCs type on disc degeneration at same and nearby cephalad levels in cervical spine. Therefore, further studies are required in future.

In total, the findings of this study are clinically significant. Our findings showed that MCs increased DD at same and nearby cephalad levels in cervical spine, indicating that MCs may be an important sign of DD at the same and nearby cephalad levels.

## Data Availability

The datasets used and analyzed during the current study are available from the corresponding author on reasonable request.

## Abbreviations

MCs: Modic changes
MRI: Magnetic resonance imaging
DD: Disc degeneration
